# Geometry-Constrained Multi-Frame Character Association for License Plate Recognition on Moving Cameras

**DOI:** 10.3390/s26185704

**Published:** 2026-09-08

**Authors:** Ufuk Asil, İlker Yoncacı

**Affiliations:** Department of Software Engineering, OSTİM Technical University, Ankara 06374, Türkiye; ilker.yoncaci@ostimteknik.edu.tr

**Keywords:** automatic license plate recognition, multi-frame fusion, video ALPR, robustness, planar geometry, moving camera, drone-mounted cameras

## Abstract

Multi-frame fusion is standard for converting frame-by-frame license plate character detections into stable, reliable readings. Character Time-Series Matching (CTM), a leading approach, associates characters across frames using the Hungarian algorithm with a fixed Euclidean distance threshold and a translation-only motion model, reporting 96.7% accuracy on the UFPR-ALPR dataset. In this work, we demonstrate that this high performance is protocol-dependent: when ground-truth static plate crops and pre-segmented tracks are used, CTM performs strongly. However, in real-world scenarios involving moving cameras (such as drone-mounted cameras, helmet-mounted cameras, and mobile platforms) where inter-frame geometry changes dynamically, baseline multi-frame association frameworks that combine fixed spatial gates with unconstrained translation propagation fail. In these cases, temporal fusion provides no benefit and degrades plate recognition performance below the single-frame baseline. Indeed, under its own Intersection over Union (IoU) tracker, this literature method correctly reads only 15.1% of plates in traffic videos recorded with a real moving camera (86 human-verified tracks). To address this vulnerability, we propose Geo-CTM (Geometry-Constrained CTM), an association pipeline integrating height-scaled adaptive matching gates, inter-frame similarity estimation via Random Sample Consensus (RANSAC), transform-guided character coasting, and co-occurrence-constrained duplicate track elimination. Systematic motion-model ablation demonstrates that while the complete association pipeline provides the primary foundation for robustness (raising mean accuracy from 85.40% to over 91.5%), estimating a similarity transform (91.82%) delivers the most physically grounded and identifiable representation on planar plates without estimation degeneration. While our method performs comparably to CTM on ideal data when using the same detector and detections, it minimizes performance loss under geometric distortion conditions where CTM is inadequate. For instance, a statistically significant improvement is achieved under a 0 → 60° perspective change; in real traffic videos, with the tracker held fixed so that the fusion layer is the only variable, performance rises from 15.1% to 26.7% under the IoU tracker of the original system and from 16.3% to 29.1% under ByteTrack (+11.6 and +12.8 points; exact McNemar p=0.021 and p=0.013), whereas changing the tracker alone while holding the fusion layer fixed moves accuracy by only 1–2 points and is not statistically significant. Finally, our error taxonomy analysis demonstrates that on the undistorted benchmark all residual errors correspond to zero-evidence cases beyond the reach of decision-level fusion, while under dynamic perspective distortion errors are dominated by association misalignment, highlighting the specific development areas that future performance improvements must target.

## 1. Introduction

Automatic License Plate Recognition (ALPR) is one of the most widely used computer vision applications in intelligent transportation systems. Covering a broad range of applications from traffic enforcement and parking management to security surveillance and highway tolling, this field possesses an extensive body of literature [1,2]. Traditional single-frame two-stage systems (license plate localization followed by character recognition) now deliver high performance levels that are near saturation [3,4,5]. In recent years, however, operational deployments have shifted from static images captured by fixed, ideally angled cameras toward dynamic moving-camera streams. Mobile platforms, such as vehicle dashcams, helmet cameras on motorcycle patrols, and handheld or aerial sensors, capture plates in dynamic scenes where viewpoint, scale, and image sharpness constantly change across frames [6,7]. Rather than relying on a single frame, integrating all visual evidence along a tracked trajectory provides a principled strategy to improve plate recognition accuracy.

**Figure 1:**
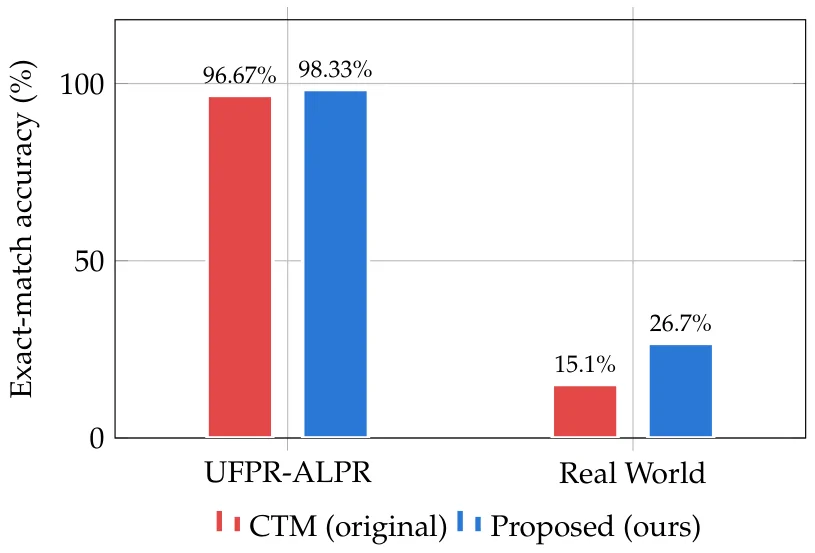
Performance comparison across controlled benchmark and real-world moving-camera regimes. **Left**: on the benchmark dataset of CTM (UFPR-ALPR test, 60 tracks, reference data crops), the two methods perform comparably: CTM achieves 96.67% (58/60; reproduced identically to the published 96.7%), while the proposed method achieves 98.33% (59/60). **Right**: when evaluated in real moving-camera traffic (86 of 87 human-verified tracks), accuracy is 15.1% versus 26.7% (+11.6 points, exact McNemar p=0.021). Both columns use the same detector, the same per-frame plate detections and the same IoU tracker—that of the original system—so only the fusion layer differs. The difference between laboratory benchmark accuracy and real-world moving-camera performance highlights the impact of the motion model assumed by the multi-frame association layer.

Multi-frame fusion directly addresses this integration challenge: multiple observations of the same plate across different frames can mutually compensate for degradations encountered in individual frames, such as motion blur, partial occlusion, or low resolution [8,9,10]. At the character level, approaches that associate and vote on frame-level detections along trajectories have reported significant improvements over single-frame methods. However, leading representatives of this paradigm typically rely on brittle association heuristics: character tracking is governed by fixed pixel-distance gates and unconstrained translation propagation. Regardless of the sophistication of the upstream multi-object tracker [11,12,13], when an association pipeline lacks adaptive spatial gating, geometrically consistent coasting, and duplicate-track handling, temporal fusion remains vulnerable to inter-frame perspective and scale variations (Figure 1).

In this study, we propose a new association and fusion model named Geo-CTM (Geometry-Constrained CTM), which enhances the Character Time-series Matching (CTM) method [14]—a prominent baseline in multi-frame ALPR—with geometric constraints. The key premise of this work is that a license plate is a rigid plane. Due to this physical property, the relative positions of characters on the plate can only vary by a planar homography transformation under camera movement relative to the plate. In scenarios where the inter-frame transformation is purely translational (for instance, a vehicle approaching a stationary toll booth in a straight line), simple association heuristics may suffice. However, in real-world scenarios such as moving vehicles, inter-frame motion inevitably involves perspective and scale changes. Under such conditions, fixed spatial thresholds and unconstrained propagation misassign character detections to incorrect tracks, causing voting evidence to accumulate destructively and dropping fusion performance even below the single-frame baseline. We support our analysis through a controlled causality protocol and extensive motion-model ablations. While our ablation reveals that the holistic association pipeline (adaptive character-height gates, transform-guided coasting, and duplicate-track elimination) provides the bulk of the robustness gain, estimating a similarity transform achieves the most principled balance of geometric fidelity and numerical stability. By applying the same distortion along a track dynamically (progressing frame-by-frame) and statically (constant across frames), we isolate when fusion hurts performance. Under static distortion, recognition accuracy declines across all methods primarily because character detector recall degrades under extreme geometric skew, a condition that decision-level temporal fusion cannot independently restore. Under dynamic distortion, the gap between the proposed geometry-constrained pipeline and baseline unconstrained translation widens. Our contributions are summarized as follows:

1. **Geometry-Constrained Character Association (Geo-CTM/G1):** We propose an association model (Geo-CTM) that estimates the inter-frame similarity transform (translation, rotation, and scale) using the Random Sample Consensus (RANSAC) algorithm. Within this model, the positions of missing characters are updated using this estimated transformation. Additionally, the matching tolerance (gate width) is scaled based on the character height instead of using a fixed pixel value. The matching process is performed in two stages: a wide-tolerance pre-matching and a narrow-tolerance reassignment. The proposed model incorporates the adaptive rotation and average translation steps of the original CTM as special cases.
2. **Co-occurrence Constrained Duplicate Track Elimination:** A co-occurrence threshold ($\tau_{\mathrm{cooc}}=0.60$) is integrated into the Union-Find track merging process. Candidate tracks that exhibit temporal co-occurrence at or above this threshold ($\mathrm{cooc}\left(T_{i},T_{j}\right)\geq \tau_{\mathrm{cooc}}$) are vetoed from merging and preserved as distinct physical characters, preventing greedy distance-based merging algorithms from erroneously subsuming adjacent characters into a single track.
3. **Experimental Demonstration of Fusion Degradation:** We develop a causality protocol that differentiates between static and dynamic distortions. We demonstrate through synthetic tests and real traffic videos that multi-frame fusion using unconstrained association and incorrect motion propagation can degrade performance rather than improve it, even dropping below the single-frame baseline accuracy.
4. **Theoretical Limits of Fusion and Error Taxonomy Analysis:** On the undistorted benchmark, we demonstrate that all residual erroneous character positions correspond strictly to zero-evidence cases where the detector never proposed the ground-truth class in any frame along the track, establishing an empirical ceiling for standalone voting-based fusion without external linguistic priors. Under progressive perspective distortion, our taxonomy additionally quantifies the emergence of vote-loss and association misalignments, revealing that geometric failure is primarily driven by association error while detector recall remains intact.

All experimental assertions in this study are established within a rigorous evaluation protocol. For a fair comparison, all evaluated methods operate on the same character detector and identical frame-level detections as inputs under a “matched and frozen detector” principle. Consequently, the observed performance discrepancies originate directly from the association and fusion layers. All operating hyperparameters of the proposed method were determined exclusively on the validation set and frozen prior to test evaluation; parameter sensitivity sweeps on the test split were conducted strictly post hoc to verify robustness across parameter neighborhoods. Statistical significance analyses are presented using the two-tailed exact binomial McNemar test. Our method is evaluated on the UFPR-ALPR [2] dataset, a hand-verified real-world traffic video, and the RodoSol-ALPR [15] dataset to synthetically measure cross-dataset generalization.

## 2. Related Work

### 2.1. Single-Frame License Plate Recognition

Traditional ALPR systems operate on single images, executing plate localization and character recognition sequentially [1]. Modern single-frame systems primarily rely on two-stage architectures built upon the YOLO detector family [3,4,5]. In particular, the layout-independent design of Laroca et al. established benchmark performance across diverse datasets [3]. These achievements are typically evaluated on benchmark datasets such as UFPR-ALPR [2] (in-vehicle cameras) and CCPD [16] (unconstrained urban scenarios). Despite high laboratory accuracy, single-frame recognition remains susceptible to transient degradations, such as motion blur, severe perspective skew, and low resolution, because it cannot draw upon temporal redundancy across video frames. In this work, we take a strong single-frame detector as a frozen baseline and overcome these limitations by combining temporal redundancy with inter-frame geometric constraints.

### 2.2. Video and Multi-Frame License Plate Recognition

The multiple appearances of a license plate throughout a video sequence naturally suggested the idea of mitigating errors by fusing these readings [8]. Approaches in the literature are highly diverse: some methods integrate detection, tracking, and recognition end-to-end into a single model [17,18], while others employ weighted voting mechanisms based on the plate’s size in the image or its confidence scores [9]. There are also practical solutions proposing to repair lost tracks using temporal interpolation [19] or selecting only the clearest frame instead of fusing all frames to reduce computational cost [20].

A prominent direction in the recent literature aggregates recognition directly over character trajectories [10]. As a representative baseline, Character Time-series Matching (CTM) tracks character detections across consecutive frames as time series [14]. However, CTM relies on an oversimplified motion assumption: it enforces a fixed Euclidean distance threshold for assignment and propagates unmatched characters using an average translation vector. While CTM includes an adaptive rotation step to mitigate static plate tilt, it lacks an explicit inter-frame planar motion model [14]. Recent formulations, such as MF-LPR2 [21], incorporate optical flow but do not formulate inter-frame transformations as explicit matching constraints. Our work addresses this gap by showing that translation-only association breaks down under moving-camera dynamics, and that an invalid motion model causes fusion to degrade recognition accuracy below the single-frame baseline.

### 2.3. Multi-Object Tracking and Association

A prerequisite for multi-frame analysis is the accurate tracking of the license plate across frames, which is directly connected to the field of multi-object tracking (MOT) [22]. The Hungarian algorithm [23] and the Kalman filter, which form the basis of tracking algorithms, were successfully combined in the SORT algorithm [11]. In subsequent years, methods such as DeepSORT [24], ByteTrack [12], and OC-SORT [13] improved tracking quality by reducing identity switches during occlusions or non-linear motions. However, the primary goal of these modern trackers is to associate the license plate with the corresponding vehicle; they do not concern themselves with the internal geometric consistency of the characters on the plate. Indeed, we observed in our experiments that, even when we replaced the tracking algorithm with a more advanced method (ByteTrack), the accuracy remained almost unchanged. This confirms that the increase in license plate recognition performance is not due to a modern tracker but stems directly from our proposed geometry-constrained character matching.

### 2.4. Geometric Correction and Robustness

The traditional way to resolve perspective distortions is to detect the license plate region and rectify it as if viewed head-on [4,25]. Additionally, deep learning-based methods that predict plate corners and rectify via homography [26,27] and geometric transformation blocks embedded within networks [28] are frequently utilized. The RANSAC algorithm has become a standard tool to clean these rectification processes of outliers [29]. However, this body of literature utilizes geometry solely to correct a single frame.

In contrast, our approach applies geometric estimation to inter-frame association rather than single-frame rectification. We estimate inter-frame motion (translation, in-plane rotation, and scale) between consecutive frames via RANSAC and bind character matching directly to this estimated transformation. On single-line plates where characters are nearly collinear, an unconstrained 8-degree-of-freedom homography produces degenerate solutions given sparse point correspondences [29,30]. Consequently, we adopt a 4-degree-of-freedom similarity transform, which provides physical fidelity while remaining numerically well-conditioned.

Furthermore, license plate recognition is fundamentally a Scene Text Recognition (STR) problem [31,32,33]. In the STR literature, static corruptions are commonly applied to evaluate model robustness [34]. Similar robustness tests are conducted in the ALPR domain for challenging weather conditions [35] or dynamic moving-camera scenarios [6]. We convert this robustness test from a static format into a dynamic one (progressing along the track) to causally reveal whether the error originates from the detector or the fusion step.

### 2.5. Literature Gap and Research Positioning

In summary: single-frame systems discard video redundancy; existing multi-frame methods assume simplistic translation models; MOT algorithms track vehicles without considering intra-plate character geometry; and rectification methods operate on single frames. Consequently, a multi-frame character fusion framework that directly enforces inter-frame planar geometry as an association constraint remains underexplored. This study addresses that gap by formulating a geometry-constrained matching pipeline that substantially reduces misassociation via RANSAC similarity estimation, coasts missing characters through the estimated transform, and remains robust under the severe perspective variations characteristic of moving-camera platforms.

## 3. Method

### 3.1. Problem Definition and Character Matching

Video-based license plate recognition systems rely on the principle of fusing multiple images of the same plate acquired throughout a video sequence to overcome the limitations of a single frame (such as instantaneous reflections or motion blur). In the first step of the system, the detections of a frozen character detector operating on frame *f* are represented as follows:

(1) $$D_{f}={\left\{\left(l_{i},c_{i},b_{i}\right)\right\}}_{i=1}^{n_{f}},\;b_{i}=\left(x_{i},y_{i},w_{i},h_{i}\right),$$

where $l_{i}\in A$ denotes the character class read by the detector, $c_{i}\in \left[0,1\right]$ is the detector’s confidence score for this reading, and $b_{i}$ represents the bounding box framing the character. We represent the center point of each detection as $p_{i}=\left(x_{i}+w_{i}/2,\;y_{i}+h_{i}/2\right)$.

The primary objective of multi-frame fusion is to associate these detection centers over time as a “track” corresponding to the same physical character. Each track undergoes majority voting to yield the most reliable character sequence [9,10].

As a prominent baseline, CTM [14] associates characters across consecutive frames using the Hungarian assignment algorithm [23]. The matching process between two frames is formulated as the following optimization:

(2) $$\min_{\pi }\;\sum_{i}\;∥p_{i}^{\left(f\right)}-p_{\pi (i)}^{\left(f+1\right)}{∥}_{2}\;\mathrm{subject}\;\mathrm{to}\;∥p_{i}^{\left(f\right)}-p_{\pi (i)}^{\left(f+1\right)}{∥}_{2}\;\leq \tau ,\;\;\tau =35\;\mathrm{px},$$

If a character is missing in the current frame (e.g., due to partial occlusion), the system projects this character into the next frame using the average translation vector (ΔC) calculated up to that frame.

However, this approach contains a structural vulnerability. Employing a fixed 35-pixel threshold and propagating missing characters solely using ΔC implicitly assumes that the camera or vehicle motion is purely translational. Yet, in real-world environments, license plates are rigid planes, and camera perspective and scale change rapidly, especially with moving cameras or maneuvering vehicles. Under such dynamic conditions, the translation-only assumption fails, leading to characters being assigned to incorrect tracks and degrading fusion performance even below the single-frame baseline. In this study, we present a geometry-constrained matching method (Geo-CTM/G1) that replaces the translation-only assumption with a more comprehensive planar motion model.

### 3.2. Geometry-Constrained Matching (G1)

To overcome this structural vulnerability of existing approaches, we model the inter-frame motion between consecutive frames using a similarity transform rather than a simple translation:

(3) $$T\left(x\right)=s\;R\;x+t,\;R=\left[\begin{array}{cc} \cos\theta & -\sin\theta \\ \sin\theta & \cos\theta \end{array}\right],$$

where s>0 represents the scale factor, R∈SO(2) represents the in-plane rotation, and $t\in R^{2}$ represents the translation; the model has a total of four degrees of freedom. In single-line license plates, characters are typically aligned along a single line; thus, a full homography with eight degrees of freedom can result in degenerate and unstable solutions given the limited number of point correspondences [29,30]. Even if the camera undergoes perspective changes (yaw/pitch), the local movement between consecutive frames primarily manifests as scale shifts and coordinate translations at the character centers. Consequently, instead of a full perspective rectification, the similarity transformation is preferred because it sufficiently models the local physical reality while avoiding excessive degrees of freedom.

The matching process consists of three main stages: *(a) Wide-Tolerance Pre-Matching:* Characters are subjected to a wide spatial gate to produce a flexible initial match ($M_{0}$). *(b) Transform Estimation via RANSAC:* Using the center coordinates of these initial matches, the transformation *T* in Equation (3) is estimated using the RANSAC algorithm [29], filtering out outlier matches. *(c) Narrow-Tolerance Reassignment:* Using the estimated transformation ($\hat{T}$), all coordinates from the first frame are projected into the second frame. Since alignment errors are minimized, a precise and final matching is performed using a tighter tolerance.

Two key mechanisms govern the proposed association model. First, instead of setting the matching tolerance as a fixed pixel value (e.g., 35 px), it is dynamically scaled according to the instantaneous character height ($\tau_{f}=\rho \;{\bar{h}}_{f}$). Consequently, as the camera approaches the plate, the matching boundaries expand proportionally. Second, we implement a hierarchical fallback strategy: if a sufficient number of character matches cannot be found to estimate the similarity matrix (which requires at least two matched pairs) or if the RANSAC reprojection error exceeds a certain threshold, the algorithm falls back first to the classical translation model ($\hat{s}=1,\hat{R}=I$), and if that also fails (i.e., zero matches), it falls back to the identity transform (no transformation). This provides a graceful fallback mechanism to protect baseline performance under adverse scenarios.

In real traffic streams, characters may not be detected in some frames due to transient occlusions or detector failures. To preserve track continuity, it is critical to predict the positions of these missing characters [17,19]. While prior approaches achieve this using the average translation vector, the proposed method projects missing character positions into the next frame using the calculated similarity transform matrix. With $p^{🟉}$ denoting the position where the character was last observed, its expected position in the new frame is expressed as:

(4) $$\hat{p}=\hat{T}\left(p^{🟉}\right)=\hat{s}\;\hat{R}\;p^{🟉}+\hat{t},$$

If we fix the scale ($\hat{s}=1$) and rotation ($\hat{R}=I$) in this equation, the projection simplifies to the translation vector used by classical approaches. Therefore, the translation-only motion model is a special case of the proposed generalized model. In real traffic conditions (e.g., vehicle approach or maneuvering), character skipping errors (such as reading 9P1926 instead of 93P192626) are largely mitigated by propagating missing character positions via the similarity transform.

A similarity transform possesses four degrees of freedom and cannot explicitly represent out-of-plane perspective deformation. We nevertheless adopt this model for a concrete empirical reason: the character centers of a single-row plate are nearly collinear, making higher-order projective models weakly identifiable. On an exactly collinear set of centers, an 8-degree-of-freedom homography can reproduce every correspondence while returning a linear part with no physical meaning—in our unit tests, it recovers a synthetic yaw warp to 0.001 px while reporting a scale of 0.06—and is rejected by the scale guard. Section 5 compares five motion models end-to-end and shows that the similarity transform achieves the highest empirical accuracy jointly with the one-dimensional projective model, while the full homography performs substantially worse.

### 3.3. Eliminating Duplicate Tracks: Co-Occurrence Constrained Merging

Minor association discrepancies during frame-by-frame matching can occasionally lead to a single physical character being represented by multiple fragmented tracks (duplicate tracks). Prior distance-only heuristics merge adjacent tracks based solely on spatial proximity, which risks erroneously conflating distinct adjacent characters into a single track.

To prevent such false merges while consolidating legitimate track fragments, we introduce a temporal co-occurrence constraint into the Union-Find merging stage. Let F(T) denote the set of video frames in which track *T* contains a character detection. For any candidate pair of tracks $\left(T_{a},T_{b}\right)$ satisfying the spatial adjacency condition, their temporal co-occurrence ratio is formally defined as:

(5) $$\mathrm{cooc}\left(T_{a},T_{b}\right)=\frac{\left|F\left(T_{a}\right)\cap F\left(T_{b}\right)\right|}{\min\left(|F\left(T_{a}\right)|,|F\left(T_{b}\right)|\right)},$$

which measures the proportion of frames in which both tracks co-exist simultaneously.

The merging decision follows a thresholding rule: tracks $T_{a}$ and $T_{b}$ are merged if and only if $\mathrm{cooc}\left(T_{a},T_{b}\right)<\tau_{\mathrm{cooc}}$, with $\tau_{\mathrm{cooc}}=0.60$. When $\mathrm{cooc}\left(T_{a},T_{b}\right)\geq \tau_{\mathrm{cooc}}$, a merge veto is enforced because the persistent simultaneous presence indicates that the tracks represent physically distinct characters. In real traffic data, distinct adjacent characters co-occur in 93–100% of frames, whereas fragmented duplicate tracks (which arise sequentially or overlap only briefly during transient detector double-detections) exhibit a co-occurrence ratio of only 23–43%. The chosen threshold of $\tau_{\mathrm{cooc}}=0.60$ cleanly separates these two regimes, preventing erroneous character merges (such as outputting 59V119335477 instead of 59V193547).

### 3.4. System Overview

The overall pipeline operates sequentially: for each consecutive frame pair, wide-tolerance pre-matching is performed, followed by RANSAC estimation of the inter-frame similarity transform. Using this estimated motion, character positions are projected to establish precise correspondences within a narrow gate. Missing characters are coasted to their predicted locations using the same transformation. Finally, fragmented tracks are consolidated via Union-Find while false merges between physically distinct characters are vetoed by the co-occurrence constraint. Measured over all 1800 frames of the UFPR test split, this process costs 0.264 ms per frame on average (standard deviation 0.079; median 0.263; p95 0.333; p99 0.520; maximum 1.480), of which the RANSAC motion estimate accounts for 0.105 ms on average (p99 0.213 ms). It is negligible compared to the processing time of character recognition models (e.g., ViTSTR [36]), which typically take 30–40 ms, and does not compromise real-time performance [9].

Algorithm 1 states the complete procedure for one plate track; every symbol it uses is listed with its value and its provenance in Table 1.

### 3.5. Limits at the Decision Stage

In addition to the geometry-constrained framework (G1), we evaluated two decision-stage extensions: frame-quality weighting based on crop resolution and Laplacian sharpness (G3), and format-constrained decoding (G2), in which the plate grammar is applied as a position-constrained argmax over the character class scores, so that a look-alike substitution such as 0→O or 1→I wins only when the accumulated evidence supports it.

However, these extensions did not yield a statistically significant improvement in the final exact-match accuracy. A detailed analysis of the undistorted test benchmark revealed a “zero-evidence” scenario in all residual failed character positions: the correct character class was never proposed by the detector in any frame along the track. When no frame provides the correct detection candidate, standalone decision-level weighting or confusion matrices operating strictly over the detector’s proposed scores cannot recover the missing information. While contextual language models, country-specific syntax constraints, or vision-language models (VLMs) may infer missing characters from linguistic priors, within a purely voting-based association pipeline, further performance improvements must directly target the character detector recall and super-resolution layers [32,33].
**Algorithm 1** Geo-CTM—geometry-constrained character fusion over one plate track.**Input:** frames $F_{1},\ldots ,F_{N}$ of one tracked plate; frozen character detector D; the constants of Table 1**Output:** the fused plate string  1:S←∅                                                               ▹ character tracks: box, votes, weights, frame set  2:**for** f=1 **to** *N* **do**  3:     $D_{f}\leftarrow M\mathrm{ERGE}B\mathrm{OXES}\left(D\left(F_{f}\right)\right)$  4:     **if** $D_{f}=\emptyset$ **then continue**                                                       ▹ the frame still counts towards *N*  5:     $w_{f}\leftarrow \sqrt{\left(h_{f}/h_{\max}\right)\;\left(\sigma_{f}/\sigma_{\max}\right)}$                                                                 ▹ G3 frame-quality weight  6:     **if** S=∅ **then**  7:           S← one new track per detection; **continue**  8:     **end if**  9:     $\bar{h}\leftarrow$ median character height in $D_{f}$10:     $M_{0}\leftarrow H\mathrm{UNGARIAN}\left(∥p_{i}-q_{j}∥\right)$, keeping pairs $\leq \max(35,\;\rho_{w}\bar{h})$                          ▹ (a) wide gate11:     $\hat{T}\leftarrow R\mathrm{ANSAC}S\mathrm{IMILARITY}\left(M_{0},\;\varepsilon =\max\left(3,\;\rho_{R}\bar{h}\right)\right)$                                        ▹ (b) motion estimate12:     **if** fewer than $k_{\min}$ inliers **or** $\hat{s}\notin \left(s_{\min},\;s_{\max}\right)$ **then**13:           $\hat{T}\leftarrow$ median translation over $M_{0}$, or the identity if $M_{0}=\emptyset$                              ▹ fallback14:     **end if**15:     $M\leftarrow H\mathrm{UNGARIAN}\left(∥\hat{T}\left(p_{i}\right)-q_{j}∥\right)$, pairs $\leq \max(10,\;\rho_{n}\bar{h})$                           ▹ (c) narrow gate16:     **for all** (i,j)∈M **do**17:           append the vote $\left(l_{j},c_{j},w_{f},f\right)$ to $S_{i}$ and set $S_{i}.\mathrm{box}\leftarrow b_{j}$18:     **end for**19:     **for all** *i* with no match in M **do**20:           $S_{i}.\mathrm{box}\leftarrow \hat{T}\left(S_{i}.\mathrm{box}\right)$, side lengths scaled by $\hat{s}$        ▹ coasting through the transform21:     **end for**22:     **for all** *j* with no match in M **do**23:           $i^{🟉}\leftarrow \arg\max_{i}\mathrm{IoU}\left(b_{j},\;S_{i}.\mathrm{box}\right)$24:           **if** $\mathrm{IoU}\left(b_{j},S_{i^{🟉}}.\mathrm{box}\right)\geq \tau_{\mathrm{new}}$ **and** $f\notin F(S_{i^{🟉}})$ **then**25:               append the vote to $S_{i^{🟉}}$                               ▹ duplicate-character rejection26:           **else**27:               open a new track from detection *j*28:           **end if**29:     **end for**30:**end for**                                       ▹ track finalization, once the plate tracker has terminated the track31:S←UNIONFINDMERGE(S): candidate pairs are those with $\Delta x<\delta_{x}\bar{w}$ and $\Delta y<\delta_{y}\bar{h}$, or $\mathrm{IoU}>\tau_{\mathrm{IoU}}$;they are visited closest first and merged **iff** $\mathrm{cooc}<\tau_{\mathrm{cooc}}$           ▹ Equation (5)32:S←{s∈S:|votes(s)|≥σN}                                                  ▹ support filter33:sort S left to right; on a two-row plate ($2{\bar{h}}_{\mathrm{plate}}>{\bar{w}}_{\mathrm{plate}}$) the upper row is read first34:for each *s*: ${\mathrm{score}}_{s}\left(a\right)\leftarrow \sum_{v\in s,\;l_{v}=a}c_{v}\;w_{v}$▹ G3 quality-weighted class scores35:**return** the position-wise argmax over ${\mathrm{score}}_{s}$ under the plate grammar                  ▹ G2

## 4. Experimental Setup

### 4.1. Datasets

The proposed geometry-constrained architecture asserts that multi-frame fusion can improve license plate recognition performance only when the underlying motion model is correctly formulated; otherwise, it can adversely affect the system. To evaluate this assertion, three distinct dataset scenarios with challenging motion dynamics were established.

We evaluate the pipeline on the UFPR-ALPR dataset, collected using vehicle-mounted moving cameras [2]. To ensure a fair and transparent comparison with previous studies in the literature, we strictly adhered to the official train/validation/test split of 60/30/60 plates (from the total 150 plates). In the second phase, to transition from controlled laboratory evaluations to real-world environments, we used a publicly available traffic recording made in Vietnam with a freely moving camera, featuring a dense flow of cars and motorcycles (URL in the Data Availability statement). Five 30 s clips were cut from it without re-encoding, starting at 00:00:40, 03:30, 05:40, 08:20 and 10:40 (1920×1080, ≈30 fps, 4505 processed frames in total); the clip times were fixed in advance to span different traffic densities and maneuver types and were not chosen by inspecting any system’s output. Track selection was automatic: every track produced by the plate detector (confidence 0.35) and followed by ByteTrack over at least five frames entered the annotation pool, giving 140 candidate tracks and 6289 plate crops, with no human pre-screening for legibility, size or difficulty. The pool is nevertheless bounded by the detector—plates the detector never found lie outside the sampling frame—so the rates we report are detector-conditional rather than absolute traffic statistics; that bound is identical for the four systems.

Ground truth was produced by verification rather than transcription. Each candidate was presented with its sharpest crop and, on demand, every frame of the track ordered by sharpness at 2× magnification, so that frames which become legible as a vehicle approaches or recedes can be read jointly. The text field was pre-filled with the reading of the baseline system—the unmodified upstream CTM association—and never with the output of the proposed method, so that any anchoring effect favors the system we compare against; the annotator, the first author (Ufuk Asil), corrected 72 of the 87 final labels (82.8%). The exclusion criterion was declared in the annotation tool before labeling began: a track no frame of which is legible to a human is discarded, because a blind guess contaminates the ground truth. This removed 53 of the 140 candidates (37.9%), leaving 87 tracks, of which 86 were matched under the trajectory-overlap association used for scoring. Discarded tracks are systematically shorter (median 15 frames against 29), so the criterion makes the resulting benchmark easier than raw traffic; it was, however, applied before any system was compared and identically to all four systems. Labeling was carried out by a single annotator, so inter-annotator agreement was not assessed; we state this as a limitation and instead release the complete annotation artifacts—the per-track crop sets, the annotation page and the resulting label file—so that the labeling can be reproduced or contested independently.

To assess generalization across different plate layouts and camera perspectives, we evaluate on the large-scale RodoSol-ALPR dataset, containing Brazilian and Mercosur formats [15]. Although this 20,000-image dataset was originally collected via stationary toll cameras, we synthetically generated dynamic video sequences by applying a software-based “dolly-in” camera approach transformation to each plate. This approach ensures that our findings are not confined to a single scenario but retain general applicability across different plate layouts.

### 4.2. Fixed and Shared Detector Protocol

In multi-frame ALPR literature, performance gains from an upgraded detector or tracker are frequently conflated with improvements in the fusion layer itself. To isolate the direct effect of the association mechanism, all experiments adhere to a shared, frozen character detector protocol. The detector employed across evaluations is YOLO11n, trained and run with the Ultralytics framework (v8.3; https://docs.ultralytics.com, accessed on 26 August 2026); its raw detections are cached and supplied identically to every compared method. Consequently, the baseline CTM algorithm and our proposed Geo-CTM method are evaluated under strictly matched detector conditions.

All compared methods—the single-frame baseline (B1), CTM association with majority voting (B2), the original CTM [14], and the proposed Geo-CTM method (B4)—receive the exact same character detector and identical raw detection results as input. The only difference among these methods is how these detections are associated across frames. Therefore, the observed accuracy discrepancies are independent of detector performance and directly reflect the efficacy of the multi-frame fusion strategy.

Indeed, the fact that the results remain virtually unchanged even when a “ByteTrack” [12] tracker is integrated under the same framework instead of IoU-based majority voting confirms that the observed performance gains originate from the RANSAC [29]-based geometric constraints built on the Hungarian assignment algorithm [23], rather than the tracking algorithm itself.

### 4.3. Hyperparameter Selection and Validation

To ensure methodological rigor and prevent data leakage, all hyperparameters were determined using an exhaustive grid search solely on the independent validation split and were frozen prior to any test-set evaluation (iou_thr =0.20, dx_ratio =0.70, dy_ratio =0.40, cooc_thr =0.60). Once the operating point was frozen based on validation performance, parameter sensitivity sweeps on the test set were conducted strictly post hoc to verify stability across parameter neighborhoods.

The grid search covers iou_thr ∈{0.20,0.25,0.30}, dx_ratio ∈{0.55,0.60,0.70,0.80} and cooc_thr ∈{0.40,0.60,0.80} with dy_ratio fixed at 0.40, and is evaluated on the 30-track UFPR validation split. All 36 grid points score identically, 25/30. The objective is flat over the entire validation grid, and the reported setting is the central point selected by a fixed tie-break rule. The full selection log is released with the code as results/hp_selection_validation.md.

Because a flat validation objective cannot by itself demonstrate that the conclusions are insensitive to the thresholds, we additionally sweep each threshold one at a time on the test split, holding the others at their frozen values, in three regimes: undistorted, the harsh end of the dynamic yaw axis, and the harsh end of the scale axis (Table 2). Three of the four thresholds change nothing at all in the undistorted and scale regimes. The duplicate-track threshold does matter under a strong perspective ramp, but as a broad plateau rather than a peak: every value from 0.30 to 0.60 gives 85.0%, and accuracy falls only above 0.70. The frozen value lies inside the plateau of all three regimes, and it was selected before any of these curves were computed.

The co-occurrence statistics—two physically distinct adjacent characters are observed together in 93–100% of the frames in which either appears, whereas the two halves of a track split by an association error co-occur in only 23–43%—provide a quantitative description of the mechanism that the veto exploits.

Furthermore, to verify the correctness of the comparison framework, we tested our replication of the CTM algorithm based on the published description and parameters from the original paper [14]. The replication yielded an exact-match accuracy of 96.67% (58/60) on the UFPR-ALPR test set, matching the reported 96.7% in the original paper exactly. This confirms the validity of the experimental setup and the objectivity of the comparison.

The four thresholds above are the only constants in the system that were chosen on data. So that the pipeline can be reproduced without reading the source, Table 1 lists every remaining numerical value as well—the two detector confidence thresholds, the wide and narrow matching gates and their character-height coefficients, the RANSAC threshold, budget and minimum inlier count, the scale guard and the fallback chain, the track termination and minimum-length rules, the support filter, and the constants of the confidence-locking mechanism—each with the origin of its value. Two points are worth stating explicitly. Values marked upstream are inherited unchanged from the baseline implementation, so they are common to every method compared here and cannot be a source of the reported differences. And the locking constants govern only the stability visualization of Section 5: every accuracy figure in this paper is produced by end-of-track voting and is independent of them.

**Code release.** The implementation, the cached per-frame detections, the evaluation log of every table in this paper and 52 unit tests are released at https://github.com/ufukasia/geo-ctm (accessed on 26 August 2026) under the MIT license, as release v1.0.0—the exact state of the code that produced the numbers reported here. The fusion core depends only on NumPy (v1.21 or later), SciPy (v1.7 or later) and OpenCV (v4.5 or later); the benchmark harness additionally needs PyTorch (v1.8 or later), torchvision (v0.9 or later) and the upstream CTM checkout, which is fetched by a setup script rather than vendored. RANSAC is the only stochastic component of the pipeline, and because the cached detections are shipped, the association and decoding stages can be re-executed on byte-identical input. We will deposit this release in Zenodo to obtain a permanent DOI for the accepted version.

### 4.4. Evaluation Metrics and Significance

Two different metrics were used to evaluate system performance. The first is exact-match accuracy, which requires the entire license plate to be read completely and without errors. This metric reflects the error-free reading requirements of real-world applications. The second is Levenshtein-distance-based character accuracy, which provides a more sensitive measurement of character-level partial errors and improvements.

Paired comparisons use the exact two-sided McNemar test. Because the samples are small (60 or 86 tracks) and the proportions sit close to one, every interval reported in this paper is exact rather than normal-approximate: accuracies carry Clopper–Pearson 95% confidence intervals (CIs), and paired comparisons carry the conditional exact interval of the odds ratio. For every primary comparison, we report the two discordant counts *b* (only the baseline correct) and *c* (only the proposed method correct), the odds ratio OR=c/b—the odds that a discordant track favors the proposed method, a direction we state explicitly because the quantity is meaningless without it—its 95% exact interval, the paired accuracy difference with its interval, and the *p*-value.

The family of comparisons used for the Bonferroni correction is declared in advance rather than chosen after the *p*-values are known. For the controlled distortion sweep, the family is the m=21 cells of the three dynamic axes, giving a corrected threshold of α/m=0.0024; for the real-traffic experiment it is the m=2 same-tracker fusion comparisons. Where a result is significant before correction but not after it, we say so: the 8 px scale cell (p=0.003) is such a case, and it is reported as suggestive rather than significant.

Two properties of the real-traffic ground truth were examined for their influence on the outcome. Nine of the 86 labels are partial transcriptions shorter than a complete Vietnamese plate; removing them leaves the conclusion unchanged and the effect slightly larger, 15.6% against 28.6% under the IoU tracker and 16.9% against 29.9% under ByteTrack, with p=0.021 in both cases. The corresponding check for vehicles that appear as more than one track is reported in Section 5.5.

### 4.5. Robustness Testing: Static and Dynamic Distortions

To push the limits of the motion model, we extended standard robustness tests [34] to the temporal domain, creating two distinct scenarios.

In the first scenario, the *Static* regime, geometric distortions such as yaw, scale change, and roll angle were applied at equal intensity across all frames of the video. While this challenges the visual limits of the character detector, it does not introduce dynamic motion changes between frames. In the second scenario, the *Dynamic* (ramp) regime, the intensity of the distortion was gradually increased along the track (for example, the camera angle was increased from 0° to 60°). This simulates the high perspective and angle changes encountered in dynamic moving cameras or during sudden maneuvers [6].

These two scenarios provide a controlled causality analysis: if the methods fail equally under both regimes, the source of the error is the visual limitation of the character detector. However, if the performance gap widens significantly only in the Dynamic regime, it provides empirical evidence that the degradation stems from the association layer’s handling of inter-frame motion dynamics (i.e., the synergy between adaptive gating, transform-guided coasting, duplicate pruning, and planar similarity modeling versus unconstrained fixed-gate translation).

### 4.6. Hardware and Computational Cost

For end-to-end evaluations, the oriented bounding box (OBB) YOLO11 plate detector was trained on an NVIDIA GeForce RTX 4060 Ti GPU (NVIDIA Corporation, Santa Clara, CA, USA). The proposed matching and fusion layer was executed on an AMD Ryzen 9 3900X CPU (Advanced Micro Devices, Inc., Santa Clara, CA, USA) to evaluate its standalone computational efficiency.

The RANSAC budget is fixed by construction—maxIters =500, confidence =0.99, without adaptive enlargement—so the computational cost is strictly bounded. Across the 1800 frames of the test split, the entire geometry-constrained matching step costs 0.264±0.079 ms per frame (p95 0.333, p99 0.520, maximum 1.480 ms). Repeating the measurement on the same tracks under a 0→60° yaw ramp, where the outlier ratio is higher, yields consistent execution time: 0.267±0.078 ms per frame, p99 0.510 ms. The similarity fit succeeds on 1739 of 1740 calls on clean frames and on 1732 of 1740 under the ramp, the remainder falling back to translation. For context, the character detector costs 10.4 ms per frame on the same CPU, so the fusion layer consumes 2.7% of the per-frame budget on average and 1.7% at the 99th percentile.

### 4.7. Detection Stage Performance

Table 3 presents the performance of the baseline models used. Our YOLO11s-OBB model, trained on the UFPR-ALPR dataset and based on OBB principles from oriented object detection research [37], achieved a 98.91% mean Average Precision at IoU=0.5 (mAP@.5) value and a 99.89% recall rate in the license plate localization task. These results are consistent with the performance of modern detection methods in the literature [38,39] and region-specific approaches [40]. The character detector (YOLO11n) trained on the RodoSol dataset obtained a 95.60% mAP@.5 value during testing.

These high localization values confirm that the accuracy gains originate from the proposed geometry-constrained multi-frame association architecture, rather than from detector performance.

## 5. Results and Analysis

### 5.1. Direct Comparison with Literature

To evaluate the proposed method’s standing in the literature, the system was directly evaluated on the UFPR-ALPR test set (Table 4). Since the earliest works in automatic license plate recognition [41], single-frame license plate recognition algorithms have progressed significantly [2,3,42]. Utilizing temporal information in video has consistently contributed to performance improvements, ranging from the work of Gonçalves et al. [43] to the CTM [14] method.

Evaluating the performance of other methods in Table 4 shows that systems combining four different models are limited to 64.9% [2], whereas more recent structures containing three models achieve 90.0% accuracy [3]. CTM, which established state-of-the-art accuracy on this benchmark, provides 91.67% exact-match accuracy on its own, and the AR + CTM configuration delivers 96.7% [14].

The proposed Geo-CTM method achieves a 98.33% accuracy rate under the identical frozen detector protocol used by AR + CTM, achieving competitive performance on this benchmark. This performance increase is achieved without incorporating an additional deep learning model and without decreasing processing speed. The fusion layer costs 0.264 ms per frame on average and 0.520 ms at the 99th percentile, against 10.4 ms per frame for the character detector on the same CPU, preserving the CTM processing speed of 47.6 frames per second (FPS).

Examining the qualitative results makes the difference between the methods clearer. Table 5 summarizes three characteristic errors of the original CTM under real traffic conditions (character dropping, character duplication, and track fragmentation) and shows how the proposed approach addresses these issues. These errors represent systematic failure modes associated with translation-only motion assumptions and unconstrained proximity-based merging. The proposed geometry-constrained association and co-occurrence veto substantially suppress these anomalies.

**Figure 2:**
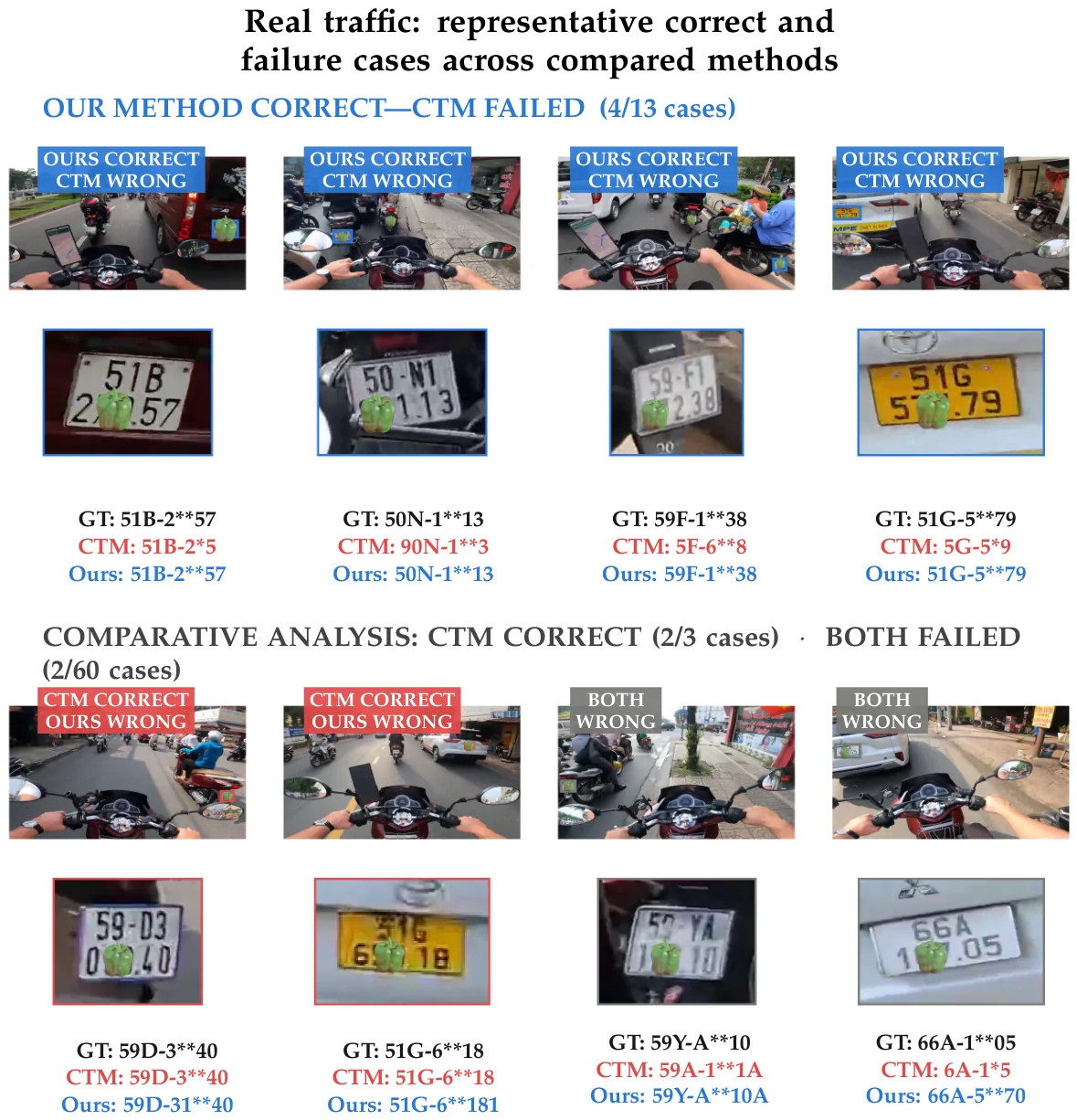
Qualitative comparison in real moving-camera traffic—an uncurated slice. (**Top row**): four scenes in which the proposed method reads correctly and the original CTM does not. (**Bottom row**): two scenes in which CTM is correct and the proposed method fails, and two in which both fail, provided for comparative analysis. The panels are an illustrative subset selected for visual clarity, not a count: the discordant-pair counts and the paired significance tests over all matched tracks are reported in Section 5.5.

Figure 2 displays frames from real Vietnam traffic where the original CTM method reads incorrectly, but the proposed method yields correct results. To provide an objective assessment, cases where Geo-CTM fails and scenes where both methods fail are reported alongside success cases in Figure 2.

Analysis of the errors of the proposed method reveals that phantom characters are generated in some cases (for example, reading 59D3103340 instead of 59D303340). This phantom digit problem occurs during rapid camera movements where motion blur causes the detector to produce duplicate bounding boxes for the same character, which our voting system interprets as a separate character. Although the duplicate track elimination method reduces these errors, it cannot completely prevent them. In the future, a simple post-processing filter tailored to country-specific plate formats can prevent such errors. In the few challenging scenarios where both methods fail, the error is caused by the poor quality of the image rather than the matching process, preventing the detector from locating the character in any frame.

### 5.2. Clean Data: Performance Equality

To analyze the performance of the proposed Geo-CTM method (B4) on clean data without geometric distortions, exact-match accuracies were measured on three different subsets of the UFPR-ALPR dataset (Table 6). The same character detector was used for all methods in the experiments.

The results show that the proposed method achieves accuracy rates of 98.33% on the test set, 83.33% on the validation set, and 96.67% on the training set. While the original CTM (B3) and IoU-based voting (B2) remain at 96.67% on the test set, the single-frame baseline without fusion (B1) achieves only 88.33% accuracy. These findings demonstrate the clear benefit of integrating temporal evidence over single-frame reading.

However, on the test set operating under ideal conditions, the difference between CTM and the proposed method is not statistically significant (p=1.0). In stable videos containing a fixed camera angle and linear motion, CTM’s simple translation assumption [14] provides sufficient performance. The main contribution of the proposed geometric constraints becomes clear in robustness and durability tests where ideal conditions are violated and camera mobility increases.

### 5.3. Robustness: Scenarios Where Fusion Helps and Hurts

**Figure 3:**
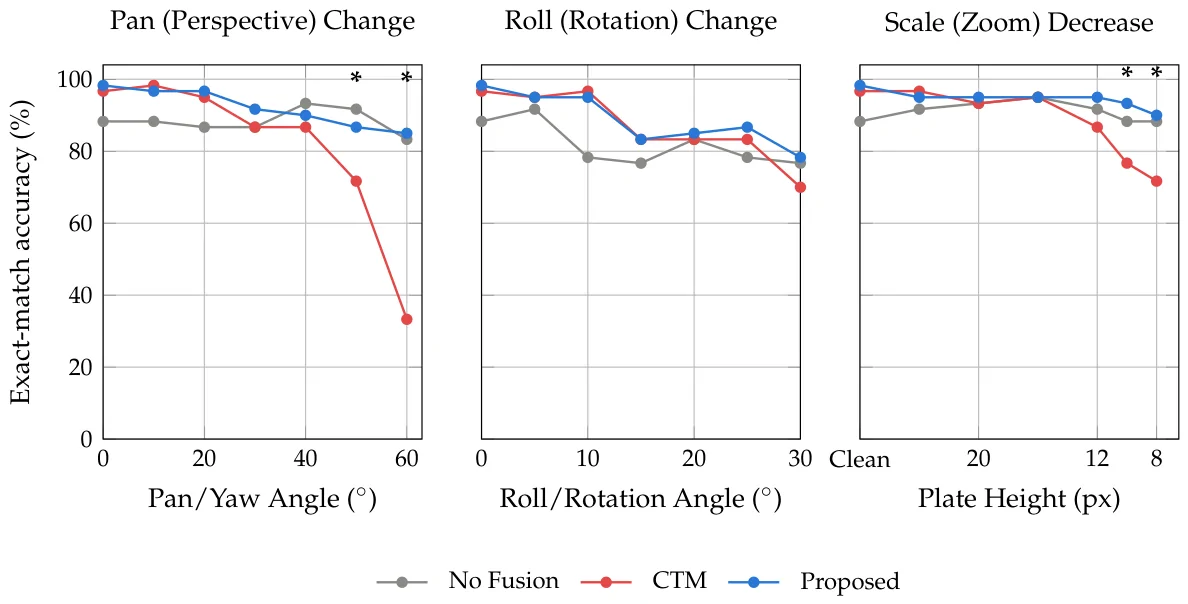
Accuracy under inter-frame geometric variations (ramp distortion applied along each track), UFPR test, 60 tracks. In the 0→60° perspective scan, CTM (red) collapses to 33.3%—dropping below the fusionless baseline (grey, 83.3%)—whereas the geometry-constrained method (blue) maintains 85.0% (+51.7 points; 0 versus 31 discordant tracks, exact McNemar $p<10^{-4}$; odds ratio in favor of the proposed method =∞, 95% exact CI [7.9,∞)). Stars mark p<0.05 (exact McNemar, uncorrected). The pre-declared family is the m=21 cells of this sweep, giving a Bonferroni threshold of α/m=0.0024; the yaw 60° and the 10 px scale cells survive it, while the 8 px cell (p=0.003) does not. The curve is B4, the proposed method (G1 + G3 + G2); the learned-confusion variant B4c is reported separately in Table 7.

The primary performance advantage of the proposed Geo-CTM pipeline emerges in dynamic moving-camera regimes where baseline association heuristics fail. To demonstrate this experimentally, ramp-type synthetic geometric distortions (with increasing intensity along the track) were added to each license plate track in the UFPR-ALPR test set, similar to the ImageNet-C methodology [34]. Figure 3 shows the results of this robustness analysis.

Particularly, when the perspective (yaw) angle reaches 60°, the baseline single-frame method yields 83.3% accuracy, while the accuracy of the original CTM algorithm degrades to 33.3%. In contrast, the proposed Geo-CTM architecture (B4) maintains an 85.0% accuracy rate (0 versus 31 discordant tracks, exact McNemar p<0.0001; odds ratio unbounded, 95% exact CI [7.9,∞)).

This demonstrates that unconstrained association heuristics and simplistic motion propagation can degrade fusion performance below single-frame accuracy by accumulating votes in incorrect character cells. As detailed in our motion-model ablation (Section 5, Table 7), this vulnerability is resolved through the complete association pipeline: dynamically scaled matching gates prevent misassignment under scale changes, transform-based coasting maintains track integrity, duplicate-track elimination prevents character fragmentation, and RANSAC-estimated similarity provides a well-conditioned geometric constraint. A robust performance was similarly obtained in the scale (zoom) variation test. In the in-plane rotation (roll) test, the two methods yield close results because CTM’s adaptive plate rotation feature can largely resolve this single degree of freedom [14]. The main advantage of our integrated framework emerges in scenarios involving complex motion, such as dynamic perspective and scale shifts.

**Figure 4:**
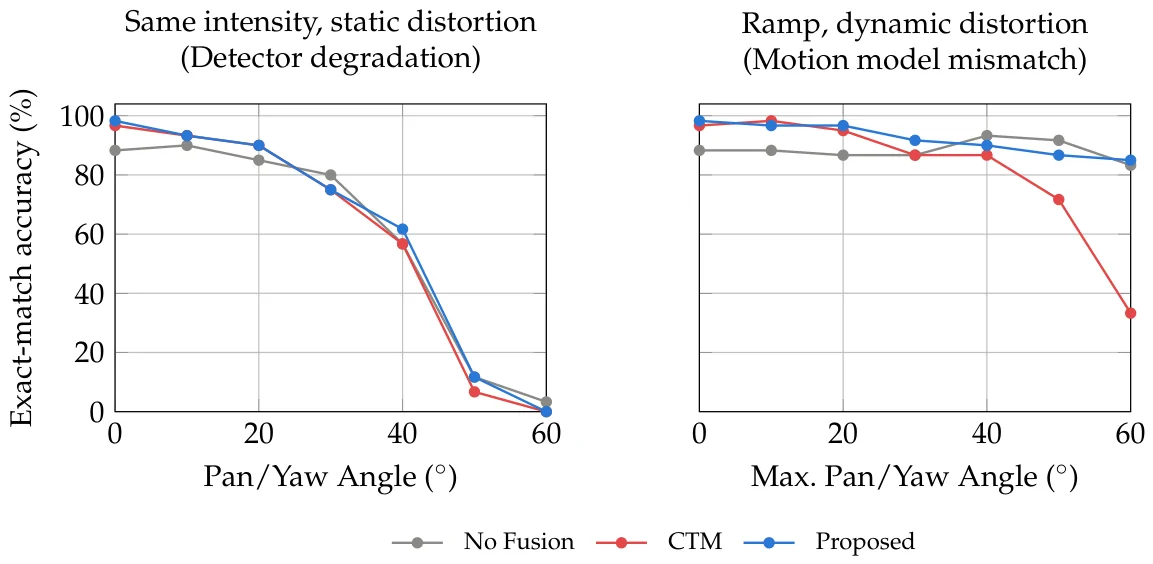
Causality control. When the same distortion is applied statically (equal across all frames), detector recall drops, and all methods degrade collectively (**left**); when it is applied dynamically (varying along the track), the translation-only motion assumption becomes inadequate, widening the performance gap in favor of the geometry-constrained association (**right**).

The origin of this performance is also visible via the causality check in Figure 4: when the distortion is applied statically and equally to all frames, all methods fail similarly due to the performance loss of the detector. However, when the distortion varies dynamically along the track, baseline CTM’s unconstrained association becomes inadequate, while the proposed pipeline preserves its performance. Our lightweight and training-free geometric association algorithm can recover much of the geometric consistency targeted by heavier optical flow or deep learning models [21] under the evaluated conditions.

### 5.4. Which Geometric Model? Five Transforms Under One Pipeline

The choice of inter-frame transform is an important consideration: yaw produces perspective distortion, and a similarity transform does not explicitly model full perspective. We, therefore, evaluated five distinct motion models inside the association step while keeping all cached per-frame detections fixed (Table 7).

Two key observations emerge. First, more degrees of freedom do not necessarily improve performance. The full homography loses 5.47 points against the similarity transform and reaches 86.35%, barely above the original CTM baseline of 85.40%: an 8-parameter model estimated from six to eight nearly collinear points is sensitive to estimation variance. The one-dimensional projective model, which incorporates its additional parameter along the plate baseline where the data provides constraint, matches the similarity transform exactly (91.82% for both across 1260 track evaluations).

Second, compact motion formulations maintain competitive accuracy: between two and six degrees of freedom the mean accuracy varies by at most 0.31 points. A translation-only model inside our pipeline still reaches 91.51%, while the original CTM—which also assumes translation—collapses to 33.3% at yaw 60° on identical detections. The robustness reported in this paper is therefore supported by the complete association pipeline: a matching gate scaled by character height instead of a fixed 35 px, missing character coasting through the estimated transform rather than a mean shift, and duplicate-track removal with a co-occurrence veto.

The similarity transform is retained because it provides a physically grounded model that remains identifiable on single-row license plate geometry without overparameterization.

### 5.5. Real World: Moving-Camera Traffic

To observe the reflection of these laboratory evaluations on real-world performance, a real Vietnam traffic video containing uncontrolled and challenging perspective angles was examined (Table 8).

This experiment was conducted under a protocol that isolates detector, tracking, and fusion effects. The full-frame plate detector is run once per clip, and its per-frame output is shared by all four systems, so every cell of Table 8 consumes byte-identical detections, and ground-truth tracks are associated with each run by trajectory overlap instead of by tracker identifier, ensuring that the evaluation does not depend on tracker-specific numeric IDs. Under this association, 86 of the 87 labeled tracks were successfully matched.

With the tracker held fixed, the original CTM system reads 15.1% of the plates under its own IoU tracker and 16.3% under ByteTrack, while the proposed Geo-CTM fusion reaches 26.7% and 29.1%, respectively. The fusion effect is therefore +11.6 points under IoU (3 versus 13 discordant tracks, odds ratio 4.33, 95% exact CI [1.19,23.71], p=0.021) and +12.8 points under ByteTrack (3 versus 14, odds ratio 4.67, [1.30,25.33], p=0.013); both survive the Bonferroni threshold for the pre-declared family of these two tests. Holding the fusion layer fixed instead and changing only the tracker moves accuracy by +1.2 points for CTM (p=1.000) and +2.3 points for Geo-CTM (p=0.754), neither significant (Table 9). The results in Figure 1 demonstrate that, despite the high accuracy of translation-only models on controlled datasets, they cannot adapt to the perspective dynamics of real moving cameras.

This experiment also provides an important empirical finding regarding the role of the tracking algorithm: with the fusion layer being held fixed, replacing the IoU tracker with ByteTrack [12] changes accuracy by +1.2 points for CTM and +2.3 points for Geo-CTM (neither statistically significant), compared to a fusion effect of +11.6 to +12.8 points that is statistically significant under either tracker. This reveals that the character association problem within a license plate cannot be solved directly by classical multi-object trackers [11,13,24]. The primary mechanism preventing duplicate characters and mismatched associations is the proposed co-occurrence veto rule, rather than the tracker itself. The co-occurrence rate of two physically distinct characters in the frames (93–100%) is statistically distinct from that of duplicate tracks split by errors (23–43%). Integrating this geometric constraint filters out incorrect merges.

### 5.6. Fusion Ceiling: Theoretical Limits at the Decision Layer

To quantify why residual errors lie beyond the reach of decision-level fusion, every character position of every failing track is assigned to exactly one class, and two ceilings are computed: the *detector ceiling*, which counts tracks in which every ground-truth class was proposed by the detector somewhere in the track, and an association ceiling, which additionally requires the fused positions to align with the ground truth (Table 10).

On the undistorted benchmark, the proposed method reaches 59/60, and the detector ceiling is also 59/60: the headroom left to decision-level fusion is 0.00 points. The single remaining failure, track0107, fails because six of its seven character positions carry zero accumulated evidence for the correct class—the detector never proposes it in any frame. No weighting scheme, confusion table or grammar acting on these candidate scores can recover it, which is also why the learned confusion prior evaluated as the B4c ablation yields no gain on this split.

The breakdown along the distortion axis is informative and supports the central thesis directly. The detector ceiling stays at 59/60 at every yaw level: under a 0→60° perspective ramp, the detector still proposes the correct character classes somewhere in the track. What degrades is the ability to put those votes in the right cell. Between 0° and 60° the association errors rise from 0 to 12 positions while accuracy falls from 59/60 to 51/60, leaving 8 tracks (13.3 points) that are recoverable in principle and lost by the fusion layer. The failure under geometric distortion is, therefore, an association failure, not a detection failure.

We also broke the taxonomy down by frame quality, using the mean Laplacian sharpness computed per frame. On the undistorted split, only one track fails, which is insufficient to support a three-way split. At yaw 60° the lowest sharpness third is read accurately (20/20) while the middle and upper thirds lose tracks (15/20 and 16/20). Frame sharpness does not predict failure in this regime; distortion level does.

This measurement also sharpens the scope of the limit itself. It is a statement about decision-level fusion over a fixed candidate set: if a class has zero accumulated score, a weighted vote cannot select it. It is not a statement that the information is unrecoverable in principle. External priors—a country-specific plate grammar, a sequence model, or a vision–language model—introduce evidence from outside this candidate set and are therefore outside the scope of the bound, not counterexamples to it.

### 5.7. End-to-End and Computational Cost

In the end-to-end experiments, the YOLO11s-OBB model, which follows the general framework of YOLO-based detectors [2] and relies on oriented bounding box principles [37], was integrated into the system for license plate localization.

The proposed method achieved a 95.00% exact-match accuracy in end-to-end test scenarios compared to CTM’s 91.67%. Another key finding from the experiments is that oriented bounding boxes (OBBs) do not offer additional contributions to the system during the plate rectification (straightening) stage (93.33%). The rectification process was performed by applying an affine transformation using the OpenCV library with the plate orientation angle to align the plate with the horizontal axis. The main reason this does not provide an additional contribution is that the character recognizer used has the capacity to read skewed characters up to a certain angle. This finding indicates that when character recognizers exhibit sufficient angular tolerance, explicit rectification modules [25,26] may offer diminishing returns relative to their computational overhead. In terms of computational cost, all proposed steps, including the RANSAC process, cost 0.264 ms per frame on average (p99 0.520 ms) and do not compromise real-time performance; the figure is unchanged under heavy distortion (0.267 ms, p99 0.510 ms).

### 5.8. Synthetic Dolly-In Generalization on RodoSol-ALPR

**Figure 5:**
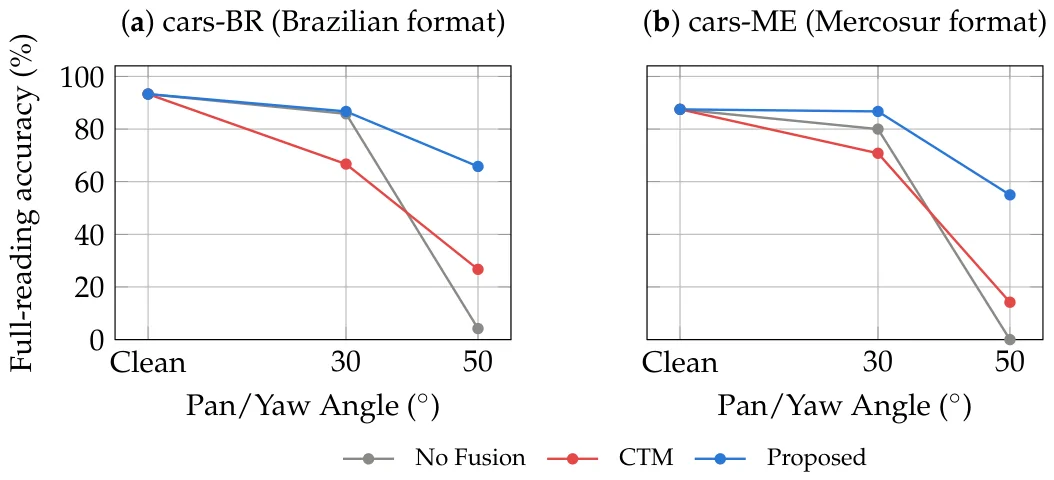
Cross-dataset generalization on RodoSol-ALPR. Under perspective distortion, the geometry-constrained method (blue) maintains recognition performance while CTM (red) degrades, across both Brazilian and Mercosur layouts on a different camera sensor.

To verify the generalizability of the proposed method to different plate formats and camera angles, experiments were also conducted on the RodoSol-ALPR dataset, which includes Brazilian and Mercosur formats [15]. In this context, video sequences were synthetically generated by applying a “dolly-in” (camera approach) movement to each plate (Table 11 and Figure 5).

Under ideal and stable conditions, all compared methods exhibited similar performance (approximately 93%). However, under challenging conditions where the perspective angle increased (50°), the accuracy of the CTM method degraded to 26.7% in the cars-BR layout and 14.2% in the cars-ME layout. The proposed method maintained accuracy rates of 65.8% and 55.0%, respectively, without requiring additional training or adaptation. These results demonstrate the generalizability of the proposed geometric constraints to different plate formats and variable camera geometries [3,15].

### 5.9. Temporal Stability and Reading Convergence

In multi-frame fusion, the goal is not only to increase the final reading accuracy but also to provide a stable plate output throughout the video stream. Continuous fluctuations of reading results across frames negatively impact system reliability.

To prevent this fluctuation, a locking mechanism was integrated that generates a dynamic confidence score based on the clarity of the video frame and character sizes [34]. A reading locks once it has repeated over at least κ=3 frames and the accumulated quality-weighted evidence reaches $E_{\mathrm{lock}}=1.6$; a locked reading is overturned only by markedly stronger contrary evidence, $E_{\mathrm{ovr}}=2.9$. The clarity score and these constants are given in the last block of Table 1. A sharp, large plate, therefore, locks within a few frames while a blurred or distant one needs many more. The mechanism serves this stability measurement alone: the accuracy figures reported elsewhere in the paper are computed by end-of-track voting and do not depend on it.

**Figure 6:**
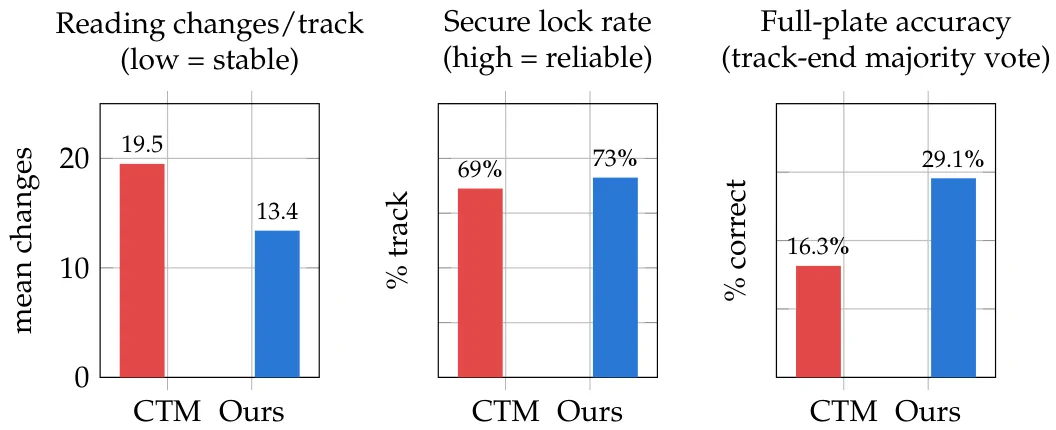
Temporal stability and accuracy (real video). Under human-like confidence-based locking (evidence weighted by frame sharpness × size), the geometry-constrained fusion produces fewer reading changes per track (less jitter), locks onto a secure reading at a higher rate, and yields higher full-plate accuracy. The lock is for visualization/stability measurement only.

As reported in Figure 6 and Table 12, the stability improvement is substantial: the original CTM method exhibits an average of 19.5 reading changes per track, whereas Geo-CTM reduces this to 13.4, representing a 31% reduction in output jitter. Both figures come from a single ByteTrack pass in which the plate boxes are identical for the two methods, so the difference is attributable to the fusion layer alone; on that same pass, the end-of-track full-plate accuracy is 29.1% against 16.3%.

## 6. Discussion, Limitations, and Conclusions

### 6.1. Discussion

The central finding of this work is that multi-frame fusion is not inherently performance-enhancing; rather, its benefit depends strictly on the robustness and physical validity of the complete character association pipeline. While the conventional ALPR paradigm assumes that temporal fusion will naturally mitigate single-frame errors [8,9,14], unconstrained distance thresholds and naive translation propagation map characters into erroneous cells, causing fusion accuracy to drop even below the single-frame baseline. This phenomenon is directly observed in moving-camera traffic (Figure 1): the original CTM system, which achieves 96.7% on static benchmark crops, drops to 15.1% in real moving-camera traffic, whereas Geo-CTM maintains 26.7% accuracy (+11.6 points, exact McNemar p=0.021) under the identical detector and tracker.

Our causality and ablation protocol isolates three key mechanisms governing this performance:

1. **Integrated Association Pipeline vs. Motion Model Expressiveness:** Our motion-model ablation (Table 7) explicitly separates the contribution of the motion model from the rest of the association pipeline. Embedding a translation-only model inside our pipeline achieves 91.51% mean accuracy across dynamic sweeps, compared to 85.40% for the baseline CTM. This demonstrates that the core mechanisms of our pipeline—character-height-scaled adaptive gates, transform-guided coasting, and co-occurrence-constrained duplicate elimination—provide the primary foundation for robustness. Upgrading from translation (91.51%) to the proposed 4-DoF similarity transform (91.82%) provides the final margin of geometric fidelity, while avoiding the severe overparameterization and estimation collapse observed in higher-order 8-DoF homographies (86.35%). Under static distortion, all methods degrade equally because character detector recall drops under extreme perspective skew, whereas under dynamic distortion, our integrated pipeline prevents destructive vote misplacement.
2. **Tracking vs. Character Association:** Upgrading the tracking algorithm from IoU to ByteTrack [12] yields negligible differences (+1.2 to +2.3 points, p≥0.75), demonstrating that object tracking and intra-plate character association require distinct geometric formulations.
3. **Duplicate Track Elimination:** The Union-Find merging rule with a co-occurrence constraint ($\tau_{\mathrm{cooc}}=0.60$) cleanly separates true adjacent characters (93–100% co-occurrence) from transient duplicate fragments (23–43%), resolving character duplication and dropping anomalies (Figure 2).

Furthermore, cross-dataset evaluations on 20,000 plates of RodoSol-ALPR confirm that the similarity constraint generalizes across diverse country layouts (Brazilian and Mercosur) without fine-tuning, while incurring a negligible runtime overhead of only 0.264 ms/frame.

### 6.2. Limitations

The limitations of this study are summarized as follows: First, while moving camera dynamics are comprehensively evaluated using real-world vehicle-mounted (UFPR-ALPR) and handheld moving-camera footage (Vietnam traffic), as well as large-scale synthetic zoom/dolly-in stress testing (RodoSol-ALPR), dedicated unmanned aerial vehicle (UAV) flight footage involves specialized aerodynamic vibrations, steep downward pitch angles (>60°), and extreme altitude transitions. Evaluating such airborne platforms remains an important avenue for future work. Additionally, robustness analysis under severe weather conditions (e.g., heavy rain, dense fog) [35] is outside the scope of this study.

Second, the zero-evidence ceiling demonstrates that, on undistorted data, characters never proposed by the detector cannot be recovered at the decision stage, whereas under dynamic distortion, association errors remain the primary target for algorithmic refinement. Third, the format rules applied are limited to the Brazilian and Vietnamese plate standards and must be adapted for other countries’ layouts.

Three further limitations concern the real-traffic evaluation: its sampling frame is bounded by the plate detector (detector-conditional rates), its ground truth was labelled by a single annotator (with all annotation artefacts released publicly), and tracker ID switches split 13 vehicles into multiple tracks (where the fusion gain survives deduplication under ByteTrack with p=0.035).

### 6.3. Conclusions

In this study, we proposed Geo-CTM, a geometry-constrained character association method that improves multi-frame license plate recognition in video streams from moving cameras. Experimental results demonstrate that an inter-frame similarity transform estimated via RANSAC protects the association pipeline from perspective and scale distortions, preventing fusion degradation.

Because decision-level fusion cannot recover classes absent from the detector proposals (the zero-evidence limit), future performance improvements should prioritize stronger visual feature representations, Transformer-based scene text recognition models [33,36], and end-to-end vision-language architectures. With optical flow-based multi-frame pixel alignment [21], ALPR systems on moving cameras and dynamic platforms will achieve heightened operational reliability.

## Figures and Tables

**Table 1:** Every numerical constant of the evaluated pipeline. $\bar{h}$ and $\bar{w}$ are the median character height and width in the current frame, *N* the number of frames in the track, and $h_{\max}$, $\sigma_{\max}$ the largest crop height and Laplacian variance along the track. *Origin* states how each value was obtained: upstream values are inherited unchanged from the baseline implementation; *validation* values were chosen by grid search on the 30-track UFPR validation split and frozen before the test split was used; *fixed* values are physical guards or budgets set once and never tuned.

Symbol	Value	Role	Origin
*Frozen front end—identical for every system in every table*			
$c_{\mathrm{plate}}$	0.35	plate detector confidence	fixed
$\iota_{\mathrm{plate}}$	0.50	plate detector NMS IoU, input 640×640	upstream
$c_{\mathrm{char}}$	0.05	character detector confidence	upstream
$\iota_{\mathrm{char}}$	0.01	character detector NMS IoU	upstream
$\iota_{\mathrm{merge}}$	0.10	IoU above which two boxes are merged into one glyph	upstream
ByteTrack	0.25/0.80/30	track activation, association threshold, lost-track buffer in frames	library default
IoU tracker	0.20/3	IoU gate and miss tolerance in frames—the baseline’s own tracker	upstream
$N_{\min}$	5 frames	shortest plate track that is read and scored	fixed
*G1—geometry-constrained matching (Algorithm 1)*			
$\rho_{w}$	$1.2\;\bar{h}$, floor 35 px	wide gate of the pre-matching stage (a)	fixed
$\rho_{n}$	$0.60\;\bar{h}$, floor 10 px	narrow gate of the reassignment stage (c)	fixed
ε	$0.30\;\bar{h}$, floor 3 px	RANSAC reprojection threshold	fixed
—	500, 0.99	RANSAC iteration budget and confidence, no adaptive enlargement	fixed
$k_{\min}$	2	inliers required to accept the similarity fit (3 for the affine and one-dimensional projective models, 4 for the homography)	minimal sample
$\left(s_{\min},s_{\max}\right)$	(0.6,1.4)	scale guard; a fit claiming a larger inter-frame scale change is discarded	fixed
η	0.60	fraction of correspondences that must reproject within ε, applied to the projective models only	fixed
fallback	sim. → transl. → id.	used when the fit is rejected; the median shift, then no motion at all	—
$\tau_{\mathrm{new}}$	0.25	IoU above which an unmatched detection joins the overlapping track instead of opening a new one, unless that track already voted in this frame	fixed
*Duplicate-track removal (Union–Find)—the only values selected on data*			
$\tau_{\mathrm{IoU}}$	0.20	IoU above which two tracks become merge candidates	validation
$\delta_{x}$	$0.70\;\bar{w}$	horizontal adjacency for merge candidacy	validation
$\delta_{y}$	$0.40\;\bar{h}$	vertical adjacency, required to keep two-row plates apart	validation
$\tau_{\mathrm{cooc}}$	0.60	merge veto of Equation (5)	validation
*Voting and decoding*			
σ	0.5N	support filter: votes a character must collect to be read	upstream
$w_{f}$	$\sqrt{\left(h_{f}/h_{\max}\right)\left(\sigma_{f}/\sigma_{\max}\right)}$, floor 0.05	G3 frame-quality weight	fixed
grammar	AAA-NNNN/none	G2 position constraint: Brazilian layout for UFPR and RodoSol, no grammar for the Vietnam plates	dataset
*Confidence locking—stability visualization of Section 5 only, not used for any accuracy figure*			
κ	3 frames	identical consecutive readings before a lock is considered	fixed
$E_{\mathrm{lock}}$, $E_{\mathrm{ovr}}$	1.6, 2.9	accumulated evidence to lock, and to overturn a lock	fixed
*q*	$\sqrt{\frac{\sigma_{f}}{\sigma_{f}+120}\cdot \frac{A_{f}}{A_{f}+9000}}$	per-frame clarity, from Laplacian variance $\sigma_{f}$ and crop area $A_{f}$	fixed

**Table 2:** Threshold sensitivity on the test split. Each threshold is swept one at a time with the others being held at their frozen values; entries are the spread of exact-match accuracy across the sweep, in percentage points, with the plateau of equal-best values in brackets. The frozen setting attains the sweep optimum in every cell. Detections are identical in every cell; only the duplicate-track thresholds change.

Threshold	Frozen	Undistorted	Yaw 0→60°	Scale 8 px
cooc_thr (0.30–0.90)	0.60	1.67 [0.50–0.90]	25.00 [0.30–0.60]	5.00 [0.30–0.80]
iou_thr (0.10–0.40)	0.20	0.00 [all]	1.67 [0.15–0.40]	0.00 [all]
dx_ratio (0.50–0.90)	0.70	0.00 [all]	1.67 [0.50–0.70]	0.00 [all]
dy_ratio (0.20–0.60)	0.40	0.00 [all]	0.00 [all]	0.00 [all]

**Table 3:** Detection stage performance (shared and frozen detectors). The license plate detector is YOLO11s-OBB, and the character detector is YOLO11n trained on RodoSol.

Stage/Model	Input	mAP@.5 (%)	Recall (%)
Plate detection (YOLO11s-OBB, UFPR)	1280	98.91	99.89 ^†^
Character detection (YOLO11n, RodoSol)	192	95.60	—

^†^ Frame-level plate detection rate (1798/1800).

**Table 4:** Direct comparison with the literature on the UFPR-ALPR test set (exact-match accuracy). Values for prior methods are taken from Che et al.; the proposed method is evaluated under an identical frozen detector protocol, reproducing the AR + CTM configuration of the same method exactly (96.67%, 58/60). Bold marks the best value in each column.

Method	Number of Models	Accuracy (%)	FPS
Laroca et al. 2018 [2]	4	64.9	—
Laroca et al. 2021 [3]	3	90.0	—
CTM (only) [14]	2	91.67	47.6
AR + CTM [14]	2	96.7	47.6
**Geo-CTM (Ours, B4)**	2	**98.33**	∼47.6

**Table 5:** Qualitative reading comparison in real traffic video. License plate strings are partially masked (*) for privacy compliance while preserving character-level error evaluation. Red marks an incorrect reading, and a check mark (✓) marks a reading that matches the ground truth exactly.

Ground-Truth	CTM (Original)	Geo-CTM
93P1**26	9P1**6 (dropping)	93P1**26 ✓
59V1**47	59V11**477 (duplication)	59V1**47 ✓
51K2**27	62**7 (track fragmentation)	51K2**27 ✓

**Table 6:** Exact-match accuracy on UFPR-ALPR (same detector, same detections; only fusion changes). On the test split, the exact (Clopper–Pearson) 95% intervals are [77.43,95.18] for B1, [88.47,99.59] for B3 and [91.06,99.96] for the proposed method, and on the 150-track pool [87.26,96.28] versus [89.76,97.67]. The paired comparison on test yields a single discordant track (0 in favor of B3, 1 in favor of ours), exact McNemar p=1.0; with one discordant pair, the odds ratio is unbounded and its 95% interval contains 1, so no effect size can be claimed from this table. Bold marks the best value in each column.

Method	Test (60)	Validation (30)	Train (60)	Pool (150)
B1 single frame (no fusion)	88.33	83.33	96.67	90.7
B2 CTM assoc. + majority vote	96.67	76.67	95.00	92.0
B3 CTM (published)	96.67	80.00	95.00	92.7
**Geo-CTM (Ours, B4)**	**98.33**	**83.33**	**96.67**	**94.7**

**Table 7:** Inter-frame motion model, compared inside the identical pipeline over the identical cached detections; only estimate_motion changes, and the fallback hierarchy below each model is the same. Entries are mean exact-match accuracy (%) over the seven levels of each dynamic axis, UFPR test, 60 tracks; the last column averages all 21 cells. B3 is unaffected by this choice and is repeated as the reference. The last row is the learned-confusion variant B4c under the similarity model for comparison. Bold marks the best value in each column.

Model	DoF	Yaw	Roll	Scale	All 21 Cells
B3 original CTM	—	81.20	86.90	88.11	85.40
Translation only	2	92.14	88.80	93.59	91.51
**Similarity (proposed)**	**4**	**92.16**	**88.80**	**94.51**	**91.82**
1-D projective (yaw)	5	91.90	88.81	94.76	91.82
Affine	6	91.90	89.27	93.36	91.51
Full homography	8	85.96	84.29	88.80	86.35
Similarity, B4c ablation	4	89.76	87.84	93.80	90.47

**Table 8:** Real-world Vietnam moving-camera traffic, human-verified ground-truth tracks. Accuracies carry exact (Clopper–Pearson) 95% intervals. The original system is the first row: CTM with its own IoU tracker. The ByteTrack rows are our upgrade of the baseline, not a result attributable to its authors. The 86 matched tracks correspond to 65 distinct plate strings, since tracker identity switches split thirteen vehicles across multiple track segments. In addition to track-level accuracy, we report a vehicle-level deduplication metric (where a vehicle is considered correctly recognized if any of its track segments is read accurately): under ByteTrack, the comparison remains significant (20.0% versus 33.8%, p=0.035), whereas under IoU, it does not (18.5% versus 27.7%, p=0.146). We retain the track as the primary operational unit because a deployed system processes every individual track, while providing the vehicle-level aggregation for complete entity-level evaluation. Bold marks the best value in each column; the red entry marks the lowest accuracy in the table, that of the original system.

Tracker	Fusion	Exact Match	Accuracy (%)	Char. Acc. (%)
IoU	CTM (original system, B3)	13/86	**15.1** [8.3, 24.5]	68.5
IoU	Geo-CTM	23/86	26.7 [17.8, 37.4]	77.6
ByteTrack	CTM	14/86	16.3 [9.2, 25.8]	69.7
ByteTrack	**Geo-CTM (B4)**	25/86	**29.1** [19.8, 39.9]	**77.6**

**Table 9:** Paired comparisons on the real-traffic set, with each factor isolated. **Upper block**: the fusion layer is varied with the tracker held fixed—this is the comparison the paper’s claim rests on. **Lower block**: the tracker is varied with the fusion layer held fixed. *b* counts tracks only the first system reads correctly, *c* only the second; the odds ratio c/b is the odds that a discordant track favours the second system, with an exact conditional 95% interval. The pre-declared family is the two fusion comparisons (α/2=0.025), which both survive.

Held Fixed	Comparison	*b*/*c*	Δ Acc. (pts)	OR [95% CI], *p*
*Fusion effect (tracker held fixed)*				
IoU tracker	CTM vs. Geo-CTM	3/13	+11.6 [+2.8, +20.4]	4.33 [1.19, 23.71], p=0.021
ByteTrack	CTM vs. Geo-CTM	3/14	+12.8 [+3.8, +21.8]	4.67 [1.30, 25.33], p=0.013
*Tracker effect (fusion held fixed)*				
CTM fusion	IoU vs. ByteTrack	3/4	+1.2 [−4.9, +7.2]	1.33 [0.23, 9.10], p=1.000
Geo-CTM fusion	IoU vs. ByteTrack	4/6	+2.3 [−4.9, +9.5]	1.50 [0.36, 7.23], p=0.754

**Table 10:** Error taxonomy and the fusion ceiling along the dynamic yaw axis (UFPR test, 60 tracks). Every character position of every failing track is assigned exactly one class. *Zero-evidence*: the ground-truth class carries zero accumulated score, so no weighted vote over these candidates can select it. *Vote-loss*: the class is present in the accumulated scores but was outvoted. *Association*: the number of fused positions does not match the ground truth. The *detector ceiling* counts tracks in which every ground-truth class was proposed somewhere in the track; it is an upper bound on any decision-level fusion over these detections.

Yaw	Proposed	Detector Ceiling	Zero-Evidence	Vote-Loss	Association
	(Tracks Correct/60)		(Character Positions)		
0°	59 (98.3%)	59 (98.3%)	6	0	0
10°	58 (96.7%)	59 (98.3%)	0	0	2
20°	58 (96.7%)	59 (98.3%)	0	0	2
30°	55 (91.7%)	59 (98.3%)	2	1	3
40°	54 (90.0%)	59 (98.3%)	4	3	5
50°	52 (86.7%)	59 (98.3%)	7	4	13
60°	51 (85.0%)	59 (98.3%)	9	3	12

**Table 11:** Synthetic “dolly-in” generalization on the RodoSol-ALPR dataset (exact-match accuracy, synthetic camera approach; 120 plates per layout). Bold marks the best value in each column.

Layout	Distortion	B1 No Fusion	B3 CTM	Geo-CTM (B4)
cars-BR	clean	93.3	93.3	**93.3**
	yaw 30°	85.8	66.7	**86.7**
	yaw 50°	4.2	26.7	**65.8**
cars-ME	clean	87.5	87.5	**87.5**
	yaw 30°	80.0	70.8	**86.7**
	yaw 50°	0.0	14.2	**55.0**

**Table 12:** Temporal stability and accuracy (real video, 86 tracks). Both methods are driven by a single ByteTrack instance: the plate boxes are identical for the two methods, and only the fusion layer differs, so this comparison is not confounded by the tracker. Lock is defined by a human-like confidence threshold (evidence weighted by frame sharpness × size) and is for representation/stability measurement only; accuracy is computed via track-end majority voting, and the original CTM fusion parameters were evaluated under the same ByteTrack detections. Bold marks the best value in each column.

Metric	CTM (Old)	Geo-CTM (B4)
Full-plate accuracy (track-end)	16.3%	**29.1%**
Reading changes/track (mean)	19.52	**13.44**
Secure lock rate	69%	**73%**
Lock time (median frame)	10	10

## Data Availability

The source code, trained model weights, and all evaluation logs (per-track, per-figure) for the developed Geo-CTM method are published at https://github.com/ufukasia/geo-ctm (accessed on 26 August 2026) (release v1.0.0, MIT licence), which is the exact version used for the experiments reported here. The public datasets used: UFPR-ALPR [2] and RodoSol-ALPR [15]. The annotation artifacts of the real-world evaluation—the per-track crop sets, the single-file annotation page and the resulting label file—are released together with the source code. The Vietnam traffic video used for real-world testing is available at https://youtu.be/3r0EEpYJFlw?si=j10Yqj1IGSYXwyRd (accessed on 26 August 2026).

## Abbreviations

The following abbreviations are used in this manuscript:

ALPR	Automatic License Plate Recognition
CTM	Character Time-series Matching
RANSAC	Random Sample Consensus
MOT	Multi-Object Tracking
IoU	Intersection over Union
OBB	Oriented Bounding Box
mAP	Mean Average Precision
FPS	Frames per Second
GT	Ground Truth
STR	Scene Text Recognition
CI	Confidence Interval
OR	Odds Ratio
VLM	Vision-Language Model
UAV	Unmanned Aerial Vehicle
PII	Personally Identifiable Information
